# Supplementary material for: Three‐dimensional modeling of flow through microvascular beds and surrounding interstitial spaces
Source: Bioeng Transl Med. 2025 Nov 14;11(1):e70085. doi: 10.1002/btm2.70085 (PMC12821219; doi:10.1002/btm2.70085)
Supplement: Supplementary file 1 — Figure S1: Discretized vascular network, vascular lumen of the network, and the interstitial space modeled as a porous domain. Figure S2: Streamlines (mm/s) of flow in the vessel network in the three control and three IL‐1β treated devices. Figure S3: Streamlines (mm/s) of flow in the vessel network in the three control and three IL‐1β treated devices. Figure S4: (a) A single channel device is used to measure the diffusive permeability of the vascular lumen. (b) Dextran (70 kDa) and fluorescent microbeads perfused through the single channel device. Figure S5: A square grid network model used for calculating the inlet pressure conditions (a) model of the square grid matrix (b) pressure and velocity heat maps of control endothelium for the grid network (c) pressure and velocity heat maps of IL1β treated endothelium for the grid network. [file BTM2-11-e70085-s001.docx]

**Three-dimensional modeling of flow through microvascular beds and surrounding interstitial spaces**

Navaneeth Krishna Rajeeva Pandian^1,2^, Alanna Farrell^1,2^, Emily Davis^1,2^, Subramanian Sundaram^1,2^, Abraham Christoffel Ignatius van Steen^1,2^, Jessica Li Chang Teo^1,2^, Jeroen Eyckmans^1,2^, and Christopher S Chen^1,2^

^1^ Harvard Wyss Institute for Biologically Inspired Engineering, Boston, MA 02115, USA

^2^ Biological Design Center, Department of Biomedical Engineering, Boston University, Boston, MA 02215, USA;

The Matlab scripts and Java codes in Comsol used in this manuscript can be downloaded from: <https://github.com/NavaneethKrishnaRP/3D-Flow-in-Microvessels---Interstitial-Spaces.git>


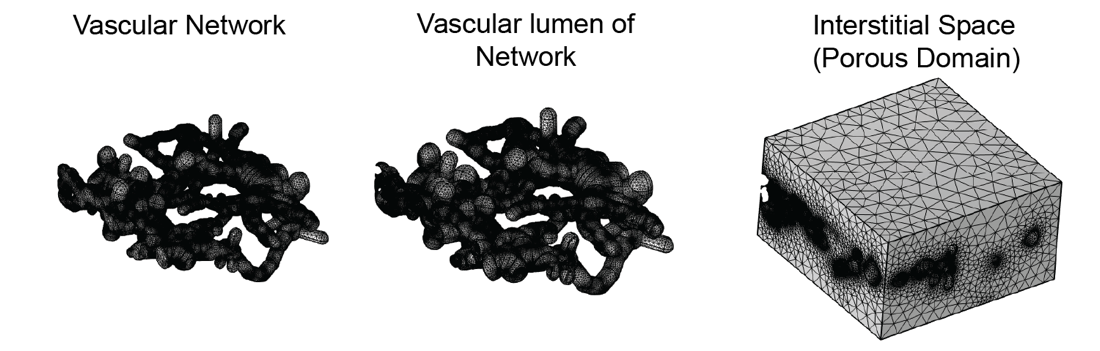


Figure S 1: Discretized vascular network, vascular lumen of the network, and the interstitial space modeled as a porous domain.


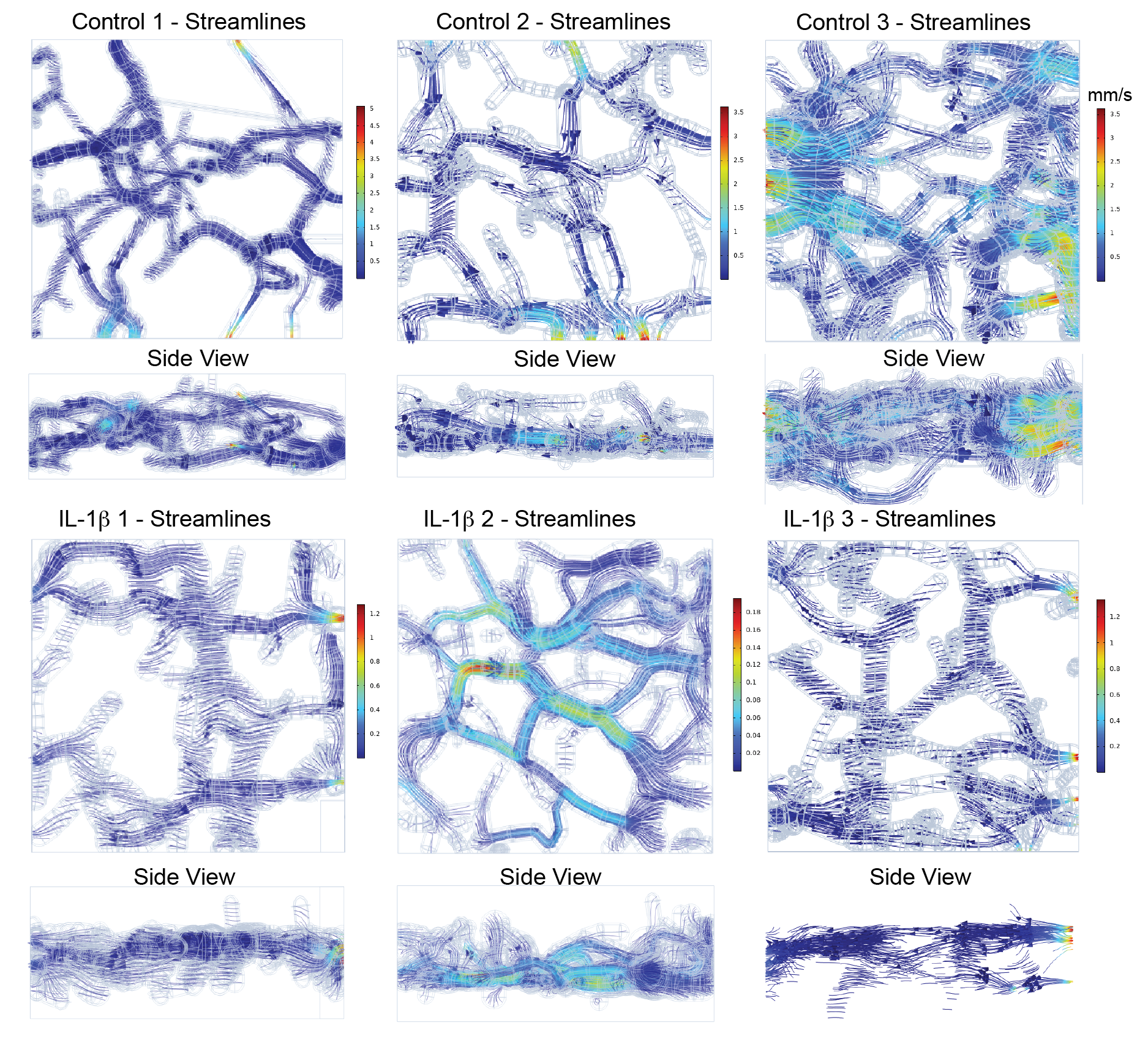


Figure S 2: Streamlines (mm/s) of flow in the vessel network in the three control and three IL-1β treated devices.


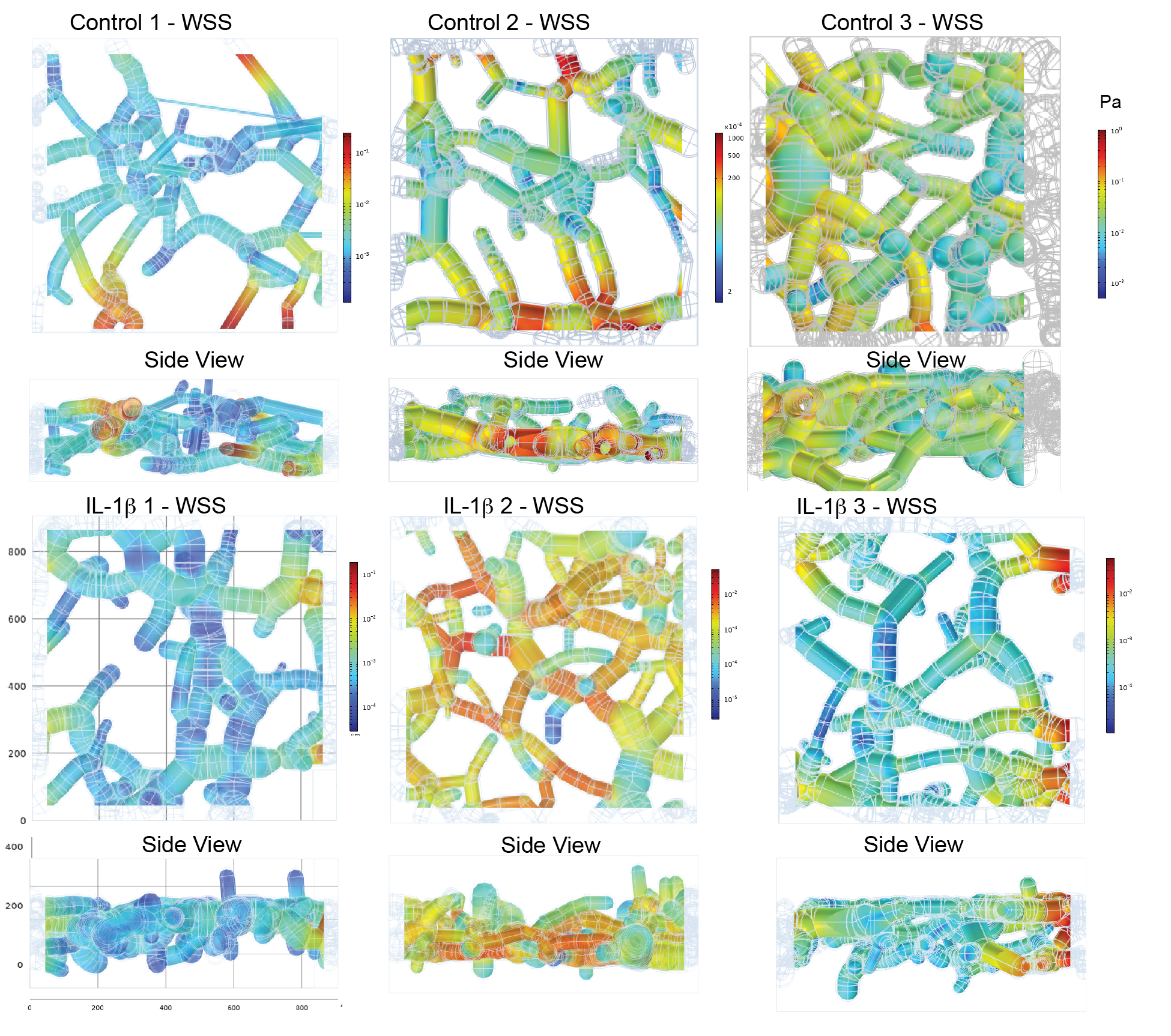


Figure S 3: Streamlines (mm/s) of flow in the vessel network in the three control and three IL-1β treated devices.


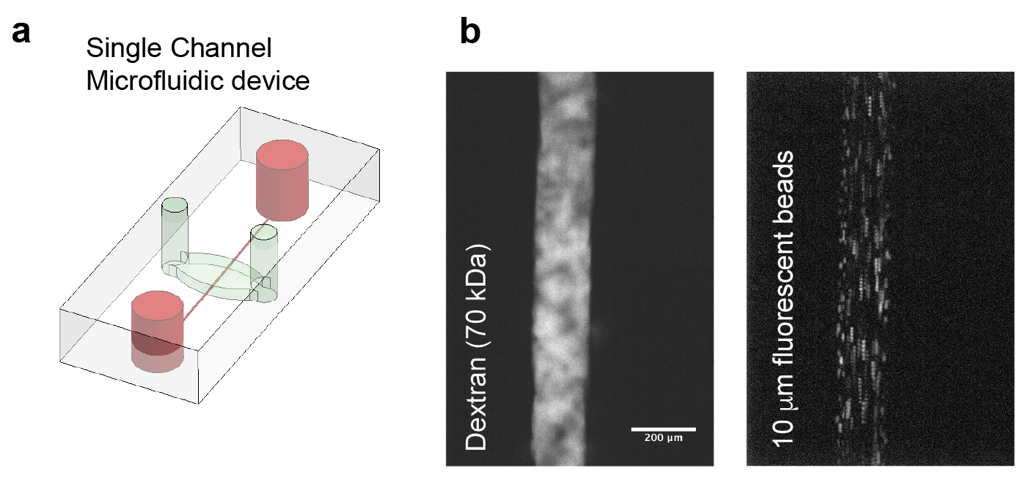


Figure S 4: a) A single channel device is used to measure the diffusive permeability of the vascular lumen. b) Dextran (70 kDa) and fluorescent microbeads perfused through the single channel device.


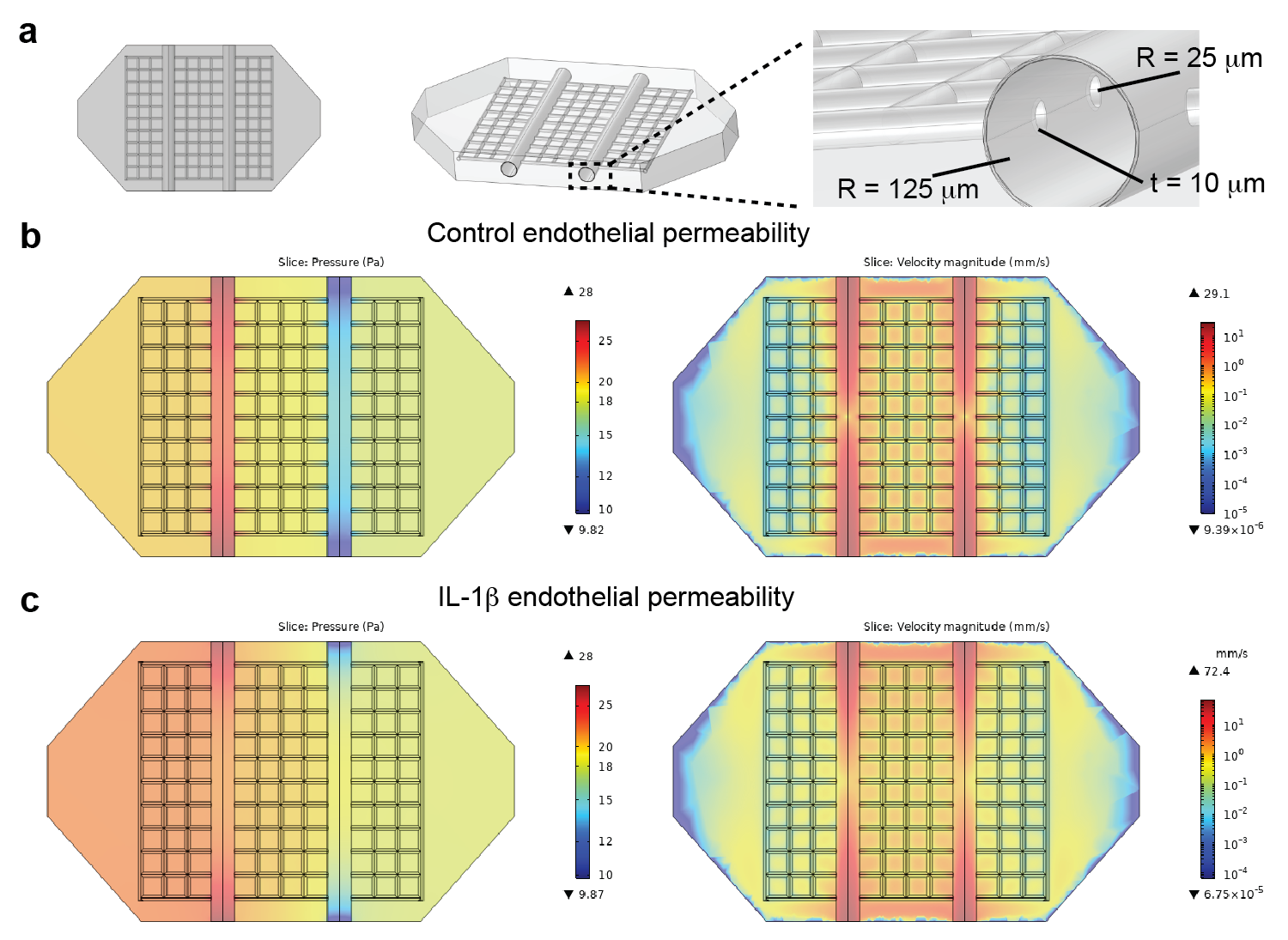


Figure S 5: A square grid network model used for calculating the inlet pressure conditions a) model of the square grid matrix b) pressure and velocity heat maps of control endothelium for the grid network c) pressure and velocity heat maps of IL1β treated endothelium for the grid network.
